# Editorial: Vitamin D Deficiency and Sufficiency in Reproduction and Bone Metabolism

**DOI:** 10.3389/fendo.2021.740021

**Published:** 2021-09-06

**Authors:** Rehana Rehman, Faiza Alam, Mukhtiar Baig, Aysha Habib Khan, Naseer Ahmed

**Affiliations:** ^1^Department of Biological & Biomedical Sciences, Aga Khan University, Karachi, Pakistan; ^2^PAPRSB Institute of Health Sciences, University of Brunei Darussalam, Bandar Seri Begawan, Brunei; ^3^Department of Clinical Biochemistry, Faculty of Medicine, Rabigh, King Abdulaziz University, Jeddah, Saudi Arabia; ^4^Department of Pathology and Laboratory Medicine, Aga Khan University, Karachi, Pakistan; ^5^Department of Medicine, Rehman Medical Institute, Peshawar, Pakistan

**Keywords:** Vitamin D, infertility, bone metabolism, follicular fluid, reproduction

Vitamin D (VD) is a steroid hormone with a well-established role in calcium metabolism, bone modeling and remodeling metabolic disorders, autoimmune conditions and reproductive system diseases (1). It is essential for maintaining mineral and skeletal internal milleu. VD is hormonally active in the form of, ‘1,25-dihydroxyvitamin D (1,25(OH)2D)’, and is responsible for its physiological actions facilitated by the VD receptor (VDR) (2). The role of VD in processes leading to reproduction is further confirmed by distribution of VD receptors (VDR) in male and female reproductive systems (3). Hypovitaminosis (less than 20ng/dL of VD) is considered a cause of insulin resistance (IR), metabolic diseases, polycystic ovary syndrome, and impaired ovarian responsiveness to assisted reproductive procedures.

An association of osteoarthritis, Type 2 Diabetes Mellitus and VD deficiency has been documented (4). This Research Topic aimed to compile recent and novel research trends on the role of VD deficiency and sufficiency in reproduction, bone and mineral diseases, VDR, VD binding proteins, association of VD with reproductive hormones, oxidative stress, infertility and impact of VD on bone, mineral health and fertility therapeutics. The articles submitted therefore focused on various aspects of bone health concerning chronic kidney disease, cholecystectomies, and female fertility during this process.

The article by Jafri et al. investigated important relationships between dietary status, anthropometric measurements, bone mass and VD status in young, healthy medical students for the first time from a lower-middle-income country (LMIC), Pakistan. It documents that lifestyle can affect bone strength, quality and peak bone mass deposit in young adults. The medical students (females) demonstrated low dietary intakes as well as insufficient bone parameters as compared to males. The data suggested that modifying one’s lifestyle throughout adolescence may assist in lowering the risk of osteoporosis and that calcaneal ultrasonography may be utilized to estimate low Bone Mineral Density (BMD). With an earlier onset of osteoporosis in Pakistan, the study has thus highlighted the importance of screening and detection of young adults who are prone to osteoporosis. Consistent delivery of public health messages on accrual of peak bone mass through a well-balanced diet and regular exercise which promotes bone development are needed. The results are supported by literature which documents that VD levels <30 ng/ml had low BMD as detected by calcaneal ultrasound; hence its role should be further explored (5).

VD deficiency is widespread in women during their reproductive lifespan. Animal studies suggest an in-depth relationship of VD with the synthesis of hormones required for reproduction. An article by Chu et al., published on our page, compared total 25(OH)D and free 25(OH)D in a sizeable group of the healthy reproductive-age women. This study observed the association of total and free 25(OH)D with ‘endocrinological, hematological and biochemical parameters. An inverse correlation of “total and free 25(OH)D with free androgen index, luteinizing hormone, testosterone, LH/FSH ratio, androstenedione, and anti-Müllerian hormone” was noticed. Similarly, inverse correlations with erythrocytes, high-sensitivity C-reactive protein (hsCRP) and the number of leukocytes and a positive relationship with few hematological parameters was observed in the study population. A significant negative correlation of low VD levels (≤30 nmol/l) with hs-CRP and WBC was reported in another study conducted by Oliveria et al. (6)

Role of VD in blastocyst formation was established by Arnanz et al. The article demonstrated an association of VD levels in follicular fluid (FF) with status of blastocyst ploidy in patients experiencing treatment for infertility. Patients with optimal levels of VD had the likelihood of attaining euploid blastocyst in comparison to patients who had VD deficiency. This suggests the role of VD in the procurement of euploid blastocysts and explains successful fertility outcomes as has been experienced by researchers (7).

A Korean countrywide population-based cohort study (Lee et al.) was also published. For this “nested case-control study”, the researchers made use of data from the “Korean National Health Insurance Corporation (NHIC) database”, containing information on nearly 97% of the Korean population. In this study, 345,940 people from the Republic of Korea, over the age of 40 years who had had a history of cholecystectomy during 2010 and 2015 were enrolled. The study aimed to evaluate fracture risk in subjects who gave history of cholecystectomy with a background relationship of low VD and BMD in post cholecystectomy patients. They concluded that individuals undergoing cholecystectomy were at a higher risk of vertebral fractures at a younger age (<50yrs). Literature supports the harmful effect of cholecystectomy on VD status; however, the mechanistic relationship of VD absorption with cholecystectomy and lower serum 25(OH)D levels needs further elaboration (8–10).

An article published in the Research Topic collection by Tariq et al. describes that VD and adipokines are involved in different metabolic processes of the body involving the bone metabolism. Resistin is a comparatively new adipokine that regulates BMD and plays a function in bone remodeling. In postmenopausal osteoporotic females, serum VD levels were lower in comparison to serum resistin levels, and VD was declared to be a negative predictor of increased serum resistin concentration. The research proposes that hypovitaminosis D is a risk factor for IR independent of adiposity (10).

In summary, this Research Topic higlighted the role of VD in bone and reproductive health, including metabolic and endocrine parameters (**Figure 1**). The presence of high levels of VD in FF highlighted its role in fertility and reproductive outcomes.

**Figure 1:**
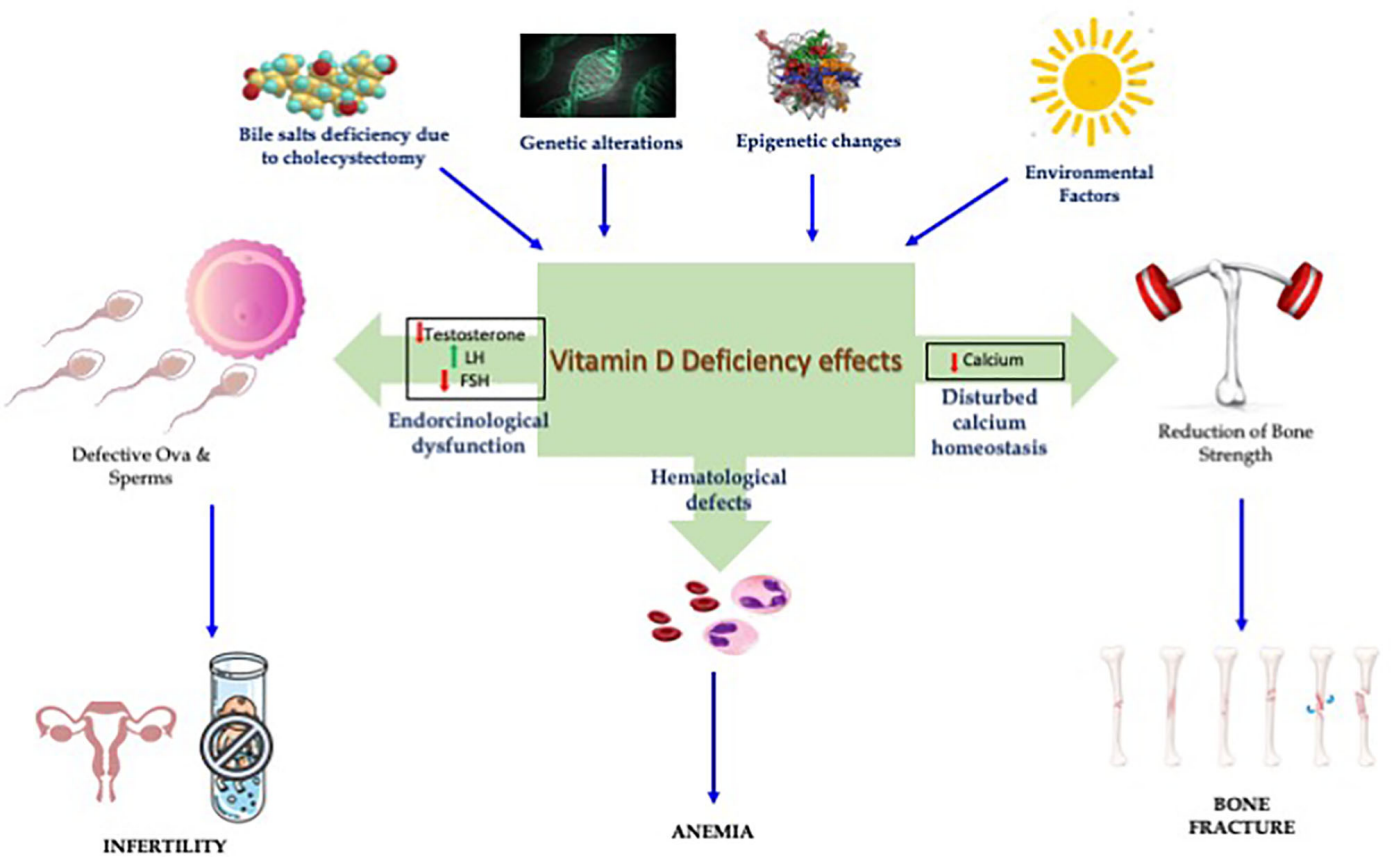
Impact of vitamin D in reproduction and bone health.

## Author Contributions

All authors reviewed and edited the manuscripts. They took part in the write up of editorial. All authors contributed to the article and approved the submitted version.

## Conflict of Interest

The authors declare that the research was conducted in the absence of any commercial or financial relationships that could be construed as a potential conflict of interest.

## Publisher’s Note

All claims expressed in this article are solely those of the authors and do not necessarily represent those of their affiliated organizations, or those of the publisher, the editors and the reviewers. Any product that may be evaluated in this article, or claim that may be made by its manufacturer, is not guaranteed or endorsed by the publisher.
